# Estrogen Receptors Mediated Negative Effects of Estrogens and Xenoestrogens in Teleost Fishes—Review

**DOI:** 10.3390/ijms23052605

**Published:** 2022-02-26

**Authors:** Konrad Wojnarowski, Paulina Cholewińska, Dušan Palić, Małgorzata Bednarska, Magdalena Jarosz, Iga Wiśniewska

**Affiliations:** 1 Chair for Fish Diseases and Fisheries Biology, Ludwig-Maximilians-University of Munich, 80539 Munich, Germany; d.palic@lmu.de; 2 Institute of Animal Breeding, Wroclaw University of Environmental and Life Sciences, 51-630 Wroclaw, Poland; paulina.cholewinska@upwr.edu.pl; 3 Department of Epizootiology and Clinic of Bird and Exotic Animals, Faculty of Veterinary Medicine, Wrocław University of Environmental and Life Sciences, 50-375 Wroclaw, Poland; malgorzata.bednarska@upwr.edu.pl (M.B.); 118685@student.upwr.edu.pl (I.W.); 4 Department of Genetics, Wroclaw University of Environmental and Life Sciences, 51-630 Wroclaw, Poland; magdalena.jarosz@upwr.edu.pl

**Keywords:** estrogens, xenoestrogens, fish, estrogen receptors, carcinogenic potential, water environment, metabolism, circulatory system

## Abstract

Estrogen receptors (ERs) play a key role in many biochemical and physiological processes, that are involved in maintaining organism homeostasis. At the most basic level, they can be divided into nuclear estrogen receptors and membrane estrogen receptors that imply their effect in two ways: slower genomic, and faster non-genomic. In these ways, estrogens and xenoestrogens can negatively affect animal health and welfare. Most of the available literature focuses on human and mammalian physiology, and clearly, we can observe a need for further research focusing on complex mutual interactions between different estrogens and xenoestrogens in aquatic animals, primarily fishes. Understanding the mechanisms of action of estrogenic compounds on the ERs in fishes and their negative consequences, may improve efforts in environmental protection of these animals and their environment and benefit society in return. In this review, we have summarized the ER-mediated effects of xenoestrogens and estrogens on teleost fishes metabolism, their carcinogenic potential, immune, circulatory, and reproductive systems.

## 1. Introduction

Estrogen receptors are proteins found in cells of many animal systems and tissues. They are commonly divided into two basic classes, nuclear receptors (nERs), which are found in the cell nucleus or in the cytoplasm, and membrane receptors (mERs), which are found in the cell membrane. Nuclear receptors such as ER-α and ER-β receptors have been studied for a while [1,2], and more recently, the GPER1 [3,4], ER-X [5,6] or Gq-mER [7,8] were identified as membrane receptors. In the case of the former, the receptor binds with the ligand through zinc fingers and the resulting complex travels to the cell nucleus, activating the so-called genomic response by initiating gene transcription, this mode of action of receptors is considered to be “slow” [9,10,11]. The second type, or membrane receptors, after binding to the ligand, act rapidly and stimulate a non-genomic response, activating intracellular signaling cascades [11,12]. Interactions of these processes and mutual influences have been reported in a manner that we can describe in accordance with Aristotle’s thought, “The whole is more than the sum of its components”.

However, teleost fishes differ from mammals by having another type of receptor ER-γ that has been first described by Paris et al. [13]. The function of ER-γ is closely related to ER-β and has been re-named as ERβ2. Such similarity between ER-β and ER-γ seems to be specific for teleost fishes likely due to a gene replication event causing the receptor’s emergence. Teleost fishes further differ from mammals by having duplicate membrane estrogen receptors *gpera* and *gperb* that probably play important role in the neuroendocrine control of reproduction [14,15]. As the name suggests, ER ligands should bind estrogens, i.e., a group of female sex hormones involved in the endocrine regulation of multiple physiological processes and a variety of animal behaviors. However, in the mid-twentieth century, scientists noticed that some natural and synthetic chemical compounds cause organisms to react almost identically to the estrogens [16,17]. Estrogenic activity of phytoestrogens in animals was first observed in 1946 in Australia, and it was linked with the “clover disease” of sheep suffering various reproduction disorders, including infertility [18].

**Figure 1:**
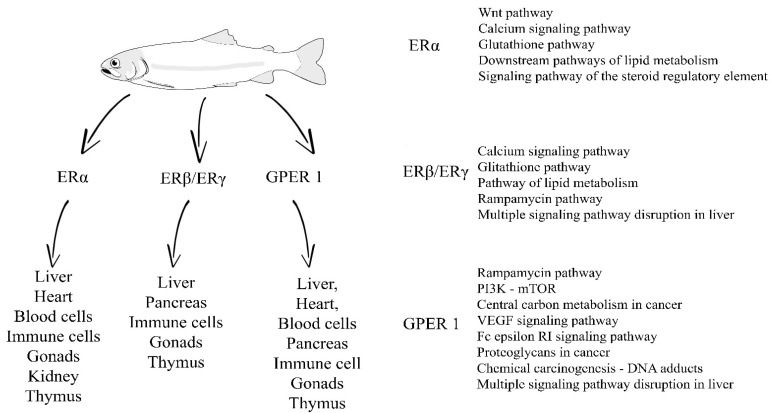
Presence of selected receptors in tissues of teleost fishes and potential disruptions of related pathways [46,47,48,49,50,51,52,53,54,55,56,57,58,59].

The following decades brought new reports on compounds that could disrupt the functioning of the endocrine system of animals. Classified as xenoestrogens, which are organic compounds that can bind or exert influence on ERs they include compounds such as polychlorinated biphenyls, bisphenols, nonylphenols, synthetic estrogens, oxybenzophenones, phytohormones and mycohormones [19,20,21,22,23,24,25,26]. During the last 15 years, there is an ongoing debate whether heavy metals and their compounds should be regarded as xenoestrogens, and if broader xenoestrogen definition should include those elements and their compounds. In this review, we will consider the broader definition of xenoestrogens that includes heavy metals [27,28,29]. Xenoestrogens influence on animal physiology is in most cases observed as having negative effects [30,31,32,33] (Figure 1). However, some of those compounds may positively affect the organism, such as anti-tumor activity of certain phytoestrogens in estrogen-dependent breast tumors [34,35,36]. The most important factor in estimating the level of risk that both hormones of natural origin and xenoestrogens will have effects on animals is the level of their presence in the environment. This is especially true for aquatic environments where detectable concentrations of endocrine disrupting chemicals, including estrogens and xenoestrogens, have long exceeded thresholds of biological activity and affected the health of many animal groups [37,38,39,40,41,42,43,44].

Animals most exposed to the presence of xenoestrogens in water are inextricably linked with the aquatic environment—teleost fishes. Despite the fact that fishes are the most numerous vertebrate class, there are gaps in our knowledge about the impact of xenoestrogens on their physiology and health [7,8,20,45]. Additionally, it should be noted that the results obtained from research on other animal groups might not always be directly translated into fish. Furthermore, the enormous diversity of fishes with over 32,000 currently known species makes it difficult to extrapolate the results of such studies to the entire class. Therefore, there is a current need to organize the available knowledge in order to support further targeted research to fill the existing gaps.

This article represents a review of our contemporary knowledge on the role of estrogen receptors and the xenoestrogenic compounds related to teleost fish health. The literature was searched between 1 October 2021 and 17 February 2022 in the Google Scholar, Web of Science and Scopus databases using the following entries: estrogen receptors, diseases, fish, Danio, Medaka, Oryzias, cardiovascular, immunology, cancer, metabolism, estrogens, xenoestrogens, reproduction, Common carp, Sea Bass, Grass Carp, Atlantic salmon, Rainbow trout, Sea bream, Nile tilapia, teleost fish.

## 2. Carcinogenic Potential

In the available literature, a substantial amount of evidence can be found for the positive therapeutic effects of endocrine-active compounds on certain neoplastic processes, such as the prevention of osteoporosis or hormone replacement [60,61,62,63]. However, multiple studies report a negative impact or even direct responsibility of such compounds for occurrences of many cancers [64,65,66,67,68]. The effects of exogenous endocrine compounds present in the aquatic environment on fish estrogen receptors are frequently characterized with negative consequences, both at population and individual levels [69,70].

The research by Lam et al. [71] demonstrated a high similarity of response pattern between zebrafish (*Danio rerio*) and human cells (MCF7, T47D and Ishikawa cells) after exposure to E2 (17—β-estradiol) (10 nM concentration). Their analysis showed that stimulation of the estrogen receptor activated highly homologous and conserved genes related to the regulation of cell cycles in zebrafish and human cells, which resulted in increased cell proliferation, DNA damage and genome instability, and weakening of the mechanisms that inhibit neoplastic processes. The obtained results also suggest the existence of similarities in the basis of the mechanism of estrogen-induced neoplasms among vertebrates and demonstrate the usefulness of zebrafish as a model organism not only in embryological or genetic studies, but also in interdisciplinary studies.

Chaturantabut et al. [72] reported that the interaction of GPER1 with E2 (10 μmol/L) in zebrafish occurs via the phosphoinositide 3-kinase-protein kinase B-mechanistic target of rapamycin pathway. They further demonstrated that GPER1, after binding with E2, is observed to develop a specific sexual dimorphism leading to the formation of hepatocellular carcinoma with a shift in the frequency of carcinogenesis processes to the zebrafish males. Furthermore, this study confirmed that a significantly increased number of GPER1 receptors was detected in HCC (hepatocellularcarcinoma) tissues, and their contact with E2 via the phos-phoinositide 3-kinase-protein kinase B-mechanistic target of rapamycin pathway and PI3K-mTOR can induce and significantly accelerate hepatocarcinogenetic processes in male zebrafish. Similar observations regarding EE2 (17-α ethinylestradiol) and the risk of neoplastic processes were shown in the case of two other fish species by Renauld et al. [46]. Using genetic and -omics methods, they showed significant multiple signaling pathway disruption in liver tissues of individuals belonging to the sardine (*Sardinops sagax*) and mackerel (*Scomber japonicas*) after short-term (5 h) exposure. Disrupted pathways included KEGG 05230 (Central carbon metabolism in cancer), KEGG 04370 (VEGF signaling pathway), KEGG 04664 (Fc epsilon RI signaling pathway), KEGG 05205 (Proteoglycans in cancer) or KEGG 05204 (Chemical carcinogenesis-DNA adducts), indicating a significantly increased risk of initiation and/or acceleration of carcinogenesis. These authors also collectively emphasized the need for further research on the impact of endocrine-active compounds on the organisms of animals and humans.

Roy et al. [73] showed that heavy metal cadmium (Cd) reacts with the zinc fingers of the zebrafish estrogen receptor, thereby replacing the zinc (Zn) atom. This substitution leads to a change in signal transduction by blocking elements of the genetic response induced by hormones in the cell nucleus [29]. Therefore, contact with Cd (Cd concentration: 0.4 mg/L) in zebrafish males leads to a phenomenon analogous to the exposure of the organism to estradiol and activation of the ERα gene in a similar way as in the observation carried out on the breast cancer cell line (MCF-7). However, in the case of females, the obtained data were not consistent with the results previously presented in the literature [74,75]. Studies conducted on the Javanese ricefish (*Oryzias javanicus*), Kim et al. [76] after treating fishes with selected concentrations of cadmium chloride CdCl_2_ (20, 40, 60, and 80 mg/L in the case of seawater and 0.25, 0.5, 1.0, 2.0 and 4.0 mg/L for freshwater) reported changes in the expression of 204 genes. These genes were responsible for multiple processes and structures, including translation, ribosomal structure, biogenesis, RNA processing and modification, cell cycle control, cell division and chromosome division. As the authors point out, such a wide range of disturbances caused by the exposure of fish to cadmium or its compounds and the association of many of them with cellular processes and important cellular pathways may constitute a risk factor potentially leading to the occurrence of neoplastic processes and immune disorders.

Meng et al. [47] in studies conducted on zebrafish showed that, similarly to estrogens, compounds such as benzophenones could act as ligands for estrogen receptors. In the case of exposure to oxybenzophenone (BP3) (4, 8, 12, 16, 20 µM concentrations), the expression of ERα, ERβ1 and GPER1 was significantly disturbed. The contact of zebrafish with BP3 also increased the expression of CYP1A and CYP1B indicating a possible role in mediation of estrogen oxidative metabolism that may cause DNA damage and disruption of Wnt pathways, and consequently, as the authors suggest, cause carcinogenesis [47]. Nonylphenols are another group of chemicals that pose a similar threat, causing significant DNA damage occurrence and leading to carcinogenesis processes after chronic exposures, similar to what has been shown in humans [77,78].

Bisphenol A (BPA) affinity for, and binding to, estrogen receptors, poses another threat to fish health [79] as it has been repeatedly potentially associated with carcinogenesis processes [79,80,81]. Cervantes-Comacho et al. [82] showed that exposure to sub-lethal doses of BPA (1, 10, 50, and 75 mg/L) caused significant DNA damage in the viviparous fish Blackfin goodea (*Goodea atripinnis*). These studies compared the expression level of Foxl2 in gonad cells and showed a statistically significant increase in fishes exposed to BPA, suggesting that prolonged exposure, among many other negative effects, may lead to increased frequency of neoplastic processes in those animals. A similar risk was identified by Major et al. [83], when each of the four endocrine active compounds studied (bifenthrin, levonorgestrel, trenbolone and EE2) had a significant negative impact on gene expression in the model species Inland silverside (*Menidia beryllina*), and led to DNA methylation and impaired cell signaling. Importantly, observed changes were multi/trans-generational. 

In studies by Teng et al. [58] performed on Nile tilapia (*Oreochromis niloticus*), they have found out that letrozole exerted its effects through the aforementioned PI3K-mTOR pathway, similarly to results obtained by Chataranbut et al. [72], thus it is highly possible that letrozole action has been mediated by ERs. As the authors have stated, growth arrest and DNA damage were observed in 1.5 μg/L concentration of letrozole and additionally affected the transcription of genes related to epigenetic regulation including UDP-glucuronosyltransferase (Ugt), glutathione S-transferase omega-1 (Gsto1), lysine-specific demethylase 6bb (Kdm6bb), jumonji and AT-rich interaction domain containing 2 (Jarid2), growth arrest and DNA damage inducible gamma (Gadd45g), and chromobox protein 7 (Cbx7).

## 3. Impact on Circulatory System

Estrogens play a key role in the proper development, maintenance, and function of the cardiovascular system of many species of vertebrates. Recently, an increased interest of scientists in the issues related to the effect of estrogens on the cardiovascular system of fish brought novel information about this link to the research community, based on advances in zebrafish transgenic model organism used in translational research in the prevention and treatment of cardiovascular disease in humans. Research by Callard et al. [84] found that the heart and blood vessels of zebrafish and goldfish (*Carassius auratus*) have similar E2 response systems that are active during their key developmental stages. Due to the significant role of estrogens in the functioning of the cardiovascular system of vertebrates, exposure of teleost fish to xenoestrogens from the aquatic environment and the potential risk of developing cardiovascular diseases become of key importance [84]. 

In studies conducted by Filice et al. [85], adult goldfish were exposed to (BPA). Individuals exposed to this compound showed impaired myocardial hemodynamics and an increase in cardiovascular indices and myocardial structural changes was observed. Further, the presence of pro-apoptotic proteins was detected in the tissues of the studied individuals. The negative effect of BPA on the circulatory system of fish is also confirmed by the studies conducted by Moreman et al. [86]. BPA significantly exerted its effect on heart tissues through estrogen receptors in a transgenic estrogen responsive zebrafish. However, in the case of the discussed studies, the negative effect of bisphenol was noted at a concentration of 2500 µg/L, thus significantly exceeding the values observed in the world’s aquatic environments. In the heart of the examined larvae, changes in the atrial:ventricular beat ratio and a significant decrease in heart rate were detected on the 14th day after fertilization. The authors claim that the risk of the presence of this compound in the aquatic environment for fishes is negligible and the significant discrepancy in the results of studies on the effects of bisphenol and xenoestrogens on the circulatory system of fishes is related to the in vitro vs. in vivo.

However, the convergence with the effect of xenoestrogens on the heart rate is indicated by Anderson et al. [48] where the significant bradycardic effect of EE2 (0.1, 1, 10, 100, and 1000 ng/L) was observed on the heart rate of the medaka embryos. In addition, using selected estrogen receptor modulators (ERMs) it has been shown that estrogen-induced bradycardia appears to be associated with GPER and not with ERα and ERβ. This study highlights GPER as a novel and alternative mode of action in terms of EE2 toxicity at environmentally relevant concentrations. On the other hand, short-term exposure of sublethal concentration (1 ppm) of BPA influences hematological parameters and biochemical changes in Mozambique tilapia (*Oreochromis mossambicus*). Exposure to sublethal BPA levels causes a reduction in the number of red blood cells (RBC), white blood cells (WBC) and hemoglobin (Hb%), which can be attributed to anemia caused by a disturbance in heme synthesis [49]. Another compound that may interact with estrogen receptors in fishes is the known mycoestrogen—Zearalenone (ZEA). In a study by Bakos et al. [87] zebrafish larvae exposed to environmentally relevant concentrations (0.1 µg/L) of ZEA developed anatomical defects of the heart muscle associated with disturbed gene expression. In studies by Orozco-Hernández et al. [59], exposition of Common carps (*Cyprinus carpio*) to the different concentrations of the E2 (1 ng/L, 1 µg/L and 1 mg/L) in water environment have induced cyto-genotoxicity effect on fish blood through influence on apoptotic signaling pathways. The cyto-genotoxic effect was manifested in an increase in the frequency of micronuclei, TUNEL-positive cells and caspase-3 activity, and the strongest effect has been observed at a highest concentration of E2.

As reported in multiple studies, negative effects of estrogenic compounds appear to be of high relevance to the juvenile or larval fish stages. It is also important to add that the complete mechanisms or pathways leading to the occurrence of circulatory system disorders or anatomical defects of its elements remain unclear.

## 4. Impact on Metabolism

Estrogens, together with their receptors (ER), play a significant role in maintaining metabolic homeostasis in the organism [88,89]. Disorders caused by ED (endocrine disruptors) including alicophenols can increase the level of estrogenic steroids in fish by inhibiting the SULT (sulfotransferase) catalyzed formation of inactive forms of estrogen sulphate. In addition, xenoestrogens, acting on estrogen receptors, can inhibit Ca-2q ATPases, leading to Ca mobilization in cells, which in turn increases the risk of apoptosis in cells and endocrine disorders in fishes [50]. The effect of substances such as benzophenones (BPs) on the regulation of the calcium signaling pathway and glutathione metabolism (GSTA, GSTM, GSTP) has also been demonstrated. Glutathione conjugation is a common estrogen detoxification mechanism and has been established as the major pathway involved in bifenthrin, trenbolone, and levonorgestrel metabolism. Additionally, changes in glutathione metabolism may contribute to epigenetic deregulation. BPs also negatively affect glucose metabolism and the amount of energy supplied to cells [47].

Membrane receptors (ERβ) binding estrogens mediate the effect of both natural estrogens and xenoestrogens on gonadotropin-stimulated 11-ketotestoserone [76]. Increasing the level of estrogen in the tissues may negatively affect the production of androgens in the testes, including gonadotropin-stimulated 11-ketotestoserone in fishes. This is probably related to a decrease in the activity of one or more steroidogenic enzymes that convert progesterone into testosterone [90].

The action of estrogens and ER is very important for autophagy in the liver, related to energy homeostasis, development, differentiation and survival. Liver autophagy is associated with multipoint ER action, including microtubule-associated protein 1A/1B-light chain 3 (LC3) nuclear transport, which is important in autophage formation and controlling excessive autophagic cell death [91,92]. The liver also synthesizes the main source of the egg yolk precursor protein, vitellogenin (Vtg), which aims to provide enough nutrients for eggs [51,52]. Its formation in the liver is related to the level of estrogen in the body, where it increases during the spawning period and decreases during non-reproduction. It also has to do with factors such as gender, time, dose, subtype and stage of reproduction. Despite recent studies, the mechanisms responsible for ERα induction by estradiol in the liver remain unclear in the majority of studied teleost spices; however, some of them indicate a significant influence of estradiol on the stabilization of ERα mRNA [51,53]. 

The liver also metabolizes lipids, which in fishes are responsible for somatic growth and gonad development. Abnormalities of lipid metabolism in fish negatively affect health, development and productivity [51,52,54]. Sun et al. [52] has demonstrated a gender-dependent pattern of lipid metabolism in zebrafish where females showed significantly higher levels of fat storage compared to males. They also had an increased level of triglycerides (TG) compared to males. The administration of E2 (200 ng/L) to females resulted in a significant increase in accumulated lipids. On the other hand, in males, administration of E2 (200 ng/L) or BPA (100 μg/L) resulted in both gonadal feminization and transformation of lipid metabolism into female phenotype [52].

The transcription of genes related to lipid metabolism is regulated directly by estrogen receptors and E2. The administration of E2 increases the expression of APOB and APOE-apolipoprotein associated with VLDL (very low-density lipoprotein) in the liver. VLDL is a lipoprotein synthesized in the liver and plays a significant role in the transport of TG. Its amount increases during the spawning period, as does the level of LPL (lipoprotein lipase)—it is responsible for the absorption of VLDL. LPL levels are significantly influenced by the amount of E2 in the body; however, there are species differences in sensitivity to it [54,93,94]. Studies conducted on fishes, mostly on zebrafish show that, inter alia, BPA increased TG, oligoglycerides, phosphatylcholine, and phosphatidylinositol during the fertilization of eggs. Mechanisms for this BPA action appear to be rather complex and related to the activation of estrogen receptors, which in turn disrupts the downstream pathways of lipid metabolism. Studies carried out to date have shown that BPA activates the signaling pathway of the steroid regulatory element binding protein-1c (srebp-1c) via the ER. In addition, it affects DNA methylation and Esrrg, where it interferes with the expression of ApoE mRNA. Overexpression of hepatic ApoE protein induced the secretion of hepatic TGs into the blood and increased their plasma levels. ApoE is produced in many tissues experiencing significant lipid flux, and the liver is the main source of ApoE synthesis in fishes, including male rare minnow (*Gobiocypris rarus*) [55]. 

Fat metabolism is also influenced by the expression level of CD36, a fatty acid transport (FA) protein that also acts as a receptor for low density oxygenated lipoproteins [87,88]. A study on triploid rainbow trout showed no change in FA translocase (FAT/CD36) expression after exposure to an increased concentration of E2, but an increase in the expression of one of the FA binding proteins (FABP)-FABP3, which binds to the aforementioned CD36. However, there was no significant increase in total FABP expression in rainbow trout after administration of E2, where the role of FABP is to oxidize or store and transport FA [95,96]. Additionally, in the studies by Zhang et al. [54] it was also shown that E2 (0 ng/L, 10 ng/L, 25 ng/L and 50 ng/L) also induced an increase in DGAT2 (last enzyme to catalyze the synthesis of triglyceride) expression in the liver of Nile tilapia. In this study, estrogen receptors were responsible for the expression of FABP3, FABP11 as well as ACO1 and LDLR related to triglyceride synthesis [83]. In a study conducted on zebrafish in 2019 by Chaturantabut et al. [72] it has been shown that inhibition of the estrogen receptor (ERβ) increased hepatocyte gene expression and blocked the effects of E2 exposure. Increasing ERβ activity also promoted epithelial differentiation activity, but at the expense of hepatocytes. 

In the studies of Celino-Brady et al. [97] the concentration of E2 (1 µg/L) and nonylphenol (NP) (10, 100 µg/L) increased the expression of insulin-like growth factor 1 (*igf1*) in the liver of Mozambique tilapia. Both E2 (1 µg/L) and NP (10 µg/L) induced 1b binding proteins in the liver (*igfbp1b*), while NP at 100 µg/L induced increased levels of 2b binding proteins (*igfbp2b*). Insulin-like growth factor plays a major role in regulating the growth of vertebrates, including fishes. Upon binding to the Gh receptor (Ghr), the release of Igf1 is stimulated, turning on the growth promoting effect in target tissues along with the promotion of a whole family of binding proteins (*igfbps*), also present in even higher variability in fishes [97]. In addition, the regulation of liver growth is also influenced by the GPER1 receptor, especially during its development, regenerative processes or neoplastic rebuilding of the liver tissues. This is due to the indirect action of GPER1 and the subsequent activation of phosphoinositide 3-kinase (PI3K): mechanistic target of rapamycin (mTOR) signaling. GPER1 promotes sex-specific liver growth in adults and, along with mTOR, is required for liver regeneration following tissue injury [51]. In the case of the GPER1 receptor, its involvement in fat metabolism or insulin secretion, as well as in the protection of pancreatic β cells has been suggested [56,57]. However, multiple metabolic functions are still under investigation.

In research by Müller et al. [98] juvenile rainbow trouts (*O. mykiss*) were exposed to the estrogenic compounds deposited in the bottom sediments of the Luppe river (Saxony, Germany). During the study, 2-, 4- and 8-fold dilutions of the sediments were used containing NP and estrone. Exposition to such a mixture of compounds has resulted in an increased abundance of transcripts of estrogen response genes and abnormal vitellogenin induction in skin mucus. The results obtained during the study show that in some circumstances bottom sediments can act as sinks for endocrine compounds and under certain conditions, significantly affect the amount of endocrine active compounds in the aquatic environment adding another layer of danger tied to the ED compounds for fish organisms.

In studies conducted by Braves et al. [99], the effects of EE2, E2 and NP (EE2: 0.004, 0.04, 0.4 nM;E2: 0.04, 0.4, 4.0 nM; NP: 4.0, 40, and 400 nM) on Atlantic salmon (*Salmo salar*) fry and smolts were assessed. As a result of exposition, EE2 exposed fry had increased Vtg mRNA levels, while igf1, igf2 and erα levels were significantly lowered; additionally, NP caused lower body mass. In smolts, hepatic Vtg mRNA was induced after 4 days of exposition, but erα was only affected by EE2 and E2. Braves et al. conclude that environmental estrogens can modulate the growth and development of the early stages of Atlantic salmon.

In studies performed by Lazaro-Velasco et al. [100], the combined effect of a low temperature of water with E2, EE2 and diethylstilbestrol on Nile tilapia has been researched. Fishes were exposed to estrogens through feed addition (120 mg/kg). As a result, fat content in the muscles of Nile tilapia was significantly higher than in control group, thus evidencing role E2 plays in the regulation of lipid metabolism in fish muscle.

As shown by cited studies, estrogens and xenoestrogens can seriously disturb many metabolic pathways and mechanisms of teleost fish. In some cases, further research is still needed to offer a full explanation of intricate mechanisms related to such disruptions.

## 5. Impact on Immune System

Estradiol can influence lymphocyte cytokine production or cytokine receptors expression and activation of effector cells. ER, especially ERα can be detected in thymocytes, bone marrow non-hematopoietic cells, T cells, B cell precursors, and circulating B cells in most of the animals, including teleost fish [101]. High expression of ERβ was detected in B cells and on almost the same level in CD8+ T cells. Multiple studies suggested that the higher concentration level of estradiol is correlated with reduced ESR1 expression, coded ERα [101,102]. ERs are differentially expressed in diverse leucocyte lineages. In vitro research based on primary cultures or freshly isolated fish macrophages and neutrophils demonstrated the influence of estradiol on chemotaxis and phagocytosis. Decreased plasma and mucus IgM was observed in rainbow trout; however, a similar decrease was not shown in Common carp [103].

The influence of E2 on the immune system of fishes is mostly observed in changes of leukocyte activities. In gilthead seabream, the number and activity of acidophilic granulocytes are correlated with the level of E2 and ER. The increase of E2 level increases the release of this cell type from the head kidney, similar to an inflammation response. Furthermore, E2 affects the transcription of several leukocyte adhesion molecules in endothelial cells thereby promoting the recruitment of acidophilic granulocytes and increasing the production of a pro-inflammatory cytokine interleukin-1β (IL1β) and inhibits acidophilic granulocytes reactive oxygen intermediates (ROIs) production activity [104]. In research by Seemann et al. [105] on sea bass, the effects of E2 (20 ng/L) on ER and cytokine gene expression measured at 90 dph (days post hatch) exposed fish delayed modification of the interleukin-1β and induced ERα gene expression. The authors further suggest that these can affect B-lymphocyte proliferation in the head kidney. Furthermore, data from 120 dph time points revealed increased phagocytic activity of head kidney monocytes/macrophages. Moreira et al. [106] reported that ER remained unchanged over the age and treatment comparisons, with the exceptions of Erβ and GPER. Both were modulated in younger cohorts and showed increased expression in the thymus of the older cohorts. The results of the above studies also indicate that proliferation/migration of the innate-like T-cell population is estrogen sensitive. The authors suggest that ERβ and GPER play key roles during thymus ontogenesis, particularly during medulla maturation.

ZEA is one of the Fusarium-produced mycotoxins whose actions are mediated through estrogen receptors. In research by Woźny et al. [107] on rainbow trout, exposure to ZEA was associated with the modulation of components of the adaptive and innate immune systems. Similar conclusions were reported by Abdel-Tawwab et al. [108] on European seabass (*Dicentrarchus labrax*). Fishes exposed to ZEA had a significant decline of total immunoglobulins in the serum after 4 weeks. Furthermore, significant down-regulation of interleukin-4 (IL-4), interleukin 1 beta (IL-1β) genes and heat shock protein 70 (HSP70) genes in the liver and anterior kidney were observed. HSP 70 plays a key role in protein metabolism and multiple cellular stress responses. The HSPs increase is connected primarily with infectious diseases, and can be observed in association with inflammatory processes triggered by environmental pollution or food toxins [109,110,111]. In another research by Abdel-Tawwab et al. [112] the ZEA (0.725 g/kg diet) toxicity suppressed the fish immunity and increased their susceptibility to *V. alginolyticus* infection causing highest mortality among all studied groups.

E2 can bind with ERs of immune cells and negatively impact immune system response, especially response towards pathogens like bacteria or parasites [105]. In a study by Rehberger et al. [113] on long term (148 days) exposure to EE2 (1.5 and 5.5 ng/L) modulated immune parameters of rainbow trout, mRNA levels of several immune genes were altered and the parasite load, as well as the disease severity, were significantly higher in EE2-exposed and infected fishes compared to infected-only control fish [104]. In another research by Kernen et al. [114] on zebrafish, the EE2 treatment reduced the thymus growth and transcript levels of thymus marker genes. Additionally, thymic estrogen receptors responded to the EE2 exposure but in a different pattern than the hepatic ER.

Studies by Liu et al. [115] have shown that in the case of Common carp, GPER1 indeed mediates the effects of BPA. The short exposure of Common carp macrophages to a 100 µg/L BPA concentration was followed by a significant increase in Ca^2+^ levels and expression of genes such as il6, il11, M17 that are related to immunotoxicity. Similar research on the effect of 17β-ethinylestradiol on juvenile Common carp was carried out by Maciuszek et al. [116]. In the in vivo part of the performed studies, fish were fed with feed with the addition of E2 (20 mg/kg) and infected with *Aeromonas salmonicida* bacteria, for in vitro part monocyte/macrophage cell suspensions were treated with 1µM E2 concentrations. Obtained results were inconsistent, and in in vitro part of studies, E2 reduced the lipopolysaccharide (LPS)-stimulated expression of pro- and anti-inflammatory mediator genes but did not affect the gene expression of the estrogen receptors and aromatase CYP19. As for in vivo in the head kidney of *A. salmonicida* infected fishes, E2-treated feeding induced an upregulation of gene expression of pro-inflammatory (il-12p35 and cxcb2) and anti-inflammatory (arginase 1, arginase 2, il-10, and mmp9) mediators. Additionally, infected fish fed with E2-treated food had increased gene expression of the estrogen receptors and aromatase CYP19. In another study by Maciuszek et al. [117] conducted on juvenile Common carps they have evaluated the effect of EE2 and 4-tert-octylphenol supplemented into fish feed (EE2 50 mg/kg, 4t-OP, 2.5 μg/day/fish). Performed studies have shown that high concentrations of EE2 and 4t-OP have induced a high number of inflammatory peritoneal leukocytes, including phagocytes and higher expression of pro-inflammatory mediators (inos, il-1β, cxcl8_l2). Additionally enhanced in vivo inflammatory reaction during bacterial infection with *A. salmonicida* has been observed. 

In studies by Kernan et al. [114], the effects of EE2 at two concentrations (3 and 10 ng/L) on zebrafish were tested. Significant thymus retardation was observed after the 64-day-long period of exposure, and transcript levels of esr1 were significantly higher compared to the control group. On the contrary, the esr2a level was significantly downregulated in EE2 exposed fishes. After the 64-day exposure, fish were introduced to the clean water and it was observed that female thymus retardation and transcripts level changes were reversible while male thymus changes and esr1 levels have not reverted to pre-exposure levels.

Discussed studies indicate that significant effects of the ERs activity can be observed in the immune system responses in teleost fishes; however, many of the aforementioned publications also suggest that our knowledge about underlying mechanisms is far from complete.

## 6. Impact on Reproductive System

As evidenced by the literature, endocrine disruptors can affect numerous other types of teleost fishes systems and tissues. Wang with co-authors [118] conducted research on the impact of ZEA added to the diet (0, 535, 1041, 1548, 2002, and 2507 μg/kg diet) on juvenile grass carps (*Ctenopharyngodon idella*). The authors have evidenced that oxidative stress, cell apoptosis, growth degradation, body deformities, necrosis of epithelial cells, blood capillary hyperemia, and goblet cell hyperplasia and formation of histopathological lesions were results of such exposure. Most likely, not all the above-mentioned effects of the ZEA are the result of the endocrine activity of this compound, but in the available literature, we can find publications suggesting that some of these negative effects are due to the ZEA impact on estrogen receptors. In studies by Pacheco Passos Neto et al. [119] they have assessed the effect of different concentrations (250 to 1000 ng/L) on Nile tilapia. Development on morphological anomalies and intersex specimens were observed, and gonads of the fishes were smaller and less developed in ZEA exposed ones. Variation in the exposure time (1–4 weeks) resulted in negative effects regardless of the exposure time. 

The influence of BPA on grass carp ovary cells has been the focus of studies by Fan et al. [120]. They have tested three concentrations: 0.3, 3, and 30 mM of BPA added to the cell culture medium (M199). In low doses creation of “a relatively reductive intracellular environment” and hormesis has been observed. In the group exposed to high (30 mM) dose effects including arrested cell proliferation, reduced cell viability, and increased cellular reactive oxygen species levels have been reported. These results support a hypothesis about crosstalk between oxidative stress and DNA methylation, which can be a key factor in understanding the cytotoxic and genotoxic mechanisms of BPA exposure. In research by Forner-Piquer et al. [121] effects of BPA on reproductive functions of gilthead sea bream (*Sparus aurata*) were studied. Two concentrations were added to the feed (4 and 4000 µg/kg of body weight). High BPA concentrations have significantly reduced sperm motility, straight-line velocity and linearity; however, there were no significant pathological alterations in gonads. On the other hand, the following reproductive markers in testis were upregulated: leptin receptor (*lepr*), estrogen receptors (*era* and *erb*), progesterone receptors (*pr*) and gonadotropin releasing hormone receptor (*gnrhr*). Additionally, BPA induced the up-regulation of the hepatic genes involved in oogenesis such as Vtg and zona pellucida 1 (*zp1*). 

Impact of E2 on growth and gonadal development of little yellow croaker (*Larimichthys polyactis*) was the topic research by Xie et al. [122] The long-term (30–90 dph) exposure to the 10 µ/L concentration of E2 has led to ovotestis, and other gonadal development retardation, and the withdrawal of E2 has not lead to the reversal of changes. Compensatory growth of the aforementioned gonads has been observed after E2 withdrawal. Due to the unique gonadal development where little yellow croaker males undergo the hermaphroditic stage, the reported results are even more significant in the terms of EDs danger to the teleost fish reproductive system.

In studies by Voisin et al. [123] mangrove killifish (*Kryptolebias marmoratus)* larvae were exposed for 28 days to 4 and 120 ng/L concentrations of EE2, correlating with disturbance of exposition proteomes in ovotestis, liver and brain. The most relevant finding was that the highest impact of EE2 on proteome was detected in the lowest concentration of EE2, 4 ng/L, noting that this concentration can be detected in many aquatic environments around the world. 

As mentioned above, heavy metals can exert endocrine disrupting effects on teleost fishes. Effects of endocrine disruption during exposure to Lead (Pb) was studied in Prussian carp (*Carassius gibelio*) by Łuszczek-Trojnar et al. [124]. Prussian carp females were exposed to Pb through feed (0, 1 (control), 8, 13, 24, and 49 mg/kg). After 12 months of exposure, the effect on LH (luteinizing hormone) has varied between the groups. In the group exposed to the lowest Pb dose (8 mg/kg), LH decreased spontaneously, and in the groups exposed to the highest two doses (24 and 48 mg/kg), a significantly higher LH secretion has been observed. After 24 months, the effect of lead exposure on LH secretion showed that the relationships were not significantly different from effects observed after 12 months, suggesting that Prussian carp females were able to adapt to the Pb exposure. In research by Paschoalini et al. [125] effects of environmental concentrations of aluminum (Al—0.2 ± 0.05 mg/mL), cadmium (Cd—not detected), copper (Cu—not detected), lead (Pb—0.002 ± 0.001 mg/mL) and iron (Fe—1.32 ± 0.05 mg/mL) on *Prochilodus argenteus* living in Paraopeba river were studied. Increased incidence of histopathological changes, as well as changes in number and morphology of germline cells, and the up-regulated expression of Vtg and zona radiata proteins in males have been observed. These results suggest that heavy metals in addition to causing tissue damage can act as estrogenic compounds in male and female *Prochilodus argenteus*.

As evidenced by research cited in this section, estrogenic endocrine disruptive compounds can seriously affect the reproductive system of many teleost fish species both on individual and at the population level, leading to the endangerment of the entire ecosystems.

## 7. Conclusions

Analysis of the available literature indicates there is a high risk that exposure to the compounds with the ability to interact with estrogen receptors may seriously interfere with fish health (Table 1). It further became obvious that presented studies have mostly focused on investigations of the single compound effects, therefore leaving gaps in our understanding of the effects of compound mixtures commonly found in the environment. Considering the multitude of impacted fish species and their considerable biological and physiological variability, differences in responses of the tested species to the tested compounds further interfere with clarity of the xenoestrogen effects. Further research on estrogen receptor mediated action of different environmentally relevant compounds and their mixtures is necessary to improve our understanding of complex genomic and non-genomic interaction mechanisms between xenoestrogens and health in aquatic animals and their habitats.

## Figures and Tables

**Table 1:** Summary of the negative effects of selected xenoestrogens and estrogens on chosen teleost fishes systems and their detected concentrations in the surface waters of the world.

Compound	Selected Negative Effects	Environmentally Detected Concentrations	References
**17-** **β-estradiol** **E2**	DNA damage Carcinogenesis Cardioviscular diseases Brachycardic effect Cyto—genetoxic effect on fish blood Lipid transformation Modification on interleukin 1β Ovotestis and gonadal development retardations;	175 ng/L—Venice lagoon/February 2002	[48,59,72,84,85,122,126]
**17-** **α** **-Ethinylestradiol** **EE2**	Carcinogenesis Neoplastic processes Hepatocarcinogenetic processes Vtg mRNA level increased Reduced Thymus growth;	21.5 ng/L—mean concentration, China, 2021	[46,72,113,127]
**Bisphenols** **BPs**	Disrupted ER expression Disrupted gene expression DNA damage Impaired myocardial hemodynamics Negative impact on blood cells (RBC, WBC, Hb%) Calcium signaling pathway Glutathione metabolism; Disrupted lipid metabolism Reduced sperm motility Morphopatological alteration in gonads Up regulated: leptin receptors, ERα and ERβ, hepatic genes and oogenesis.	63.64 ng/L New Calabar River, Nigeria, 2019	[47,49,54,55,79,80,81,82,85,121,128]
Zearaleone **ZAE**	Anatomical changes of heart muscle Disturbed gene expression Suppresed fish immune answer Negative impact on blood cells Morphological anomalies Intersex specimens;	43.7 ng/L—Poland, 2009	[87,107,112,119,129]
Nonylophenol **NP**	DNA damage Carcinogenesis Increased expression of insulin like growth factor Vtg production	106–296 ng/L—Haihe river, China, 2004	[97,98,130]
**Bifenthrin**	Disturbed gene expression Deregulation of glutathione conjugtion	51.0 mg/L—Stanislaus River, USA, 2009	[47,83,131]
**Levonorgestrel**	Disturbed gene expression Deregulation of glutathione conjugtion	0.1 ng/L—Australia, 2015	[47,83,132]
**Cadmium** **Cd**	Activation of the ERα gene Disruption of expresion of genes responsible for ribosomal structure, biogenesis, RNA processing and modification, cell cycle control, cell division and chromosome division	45.01 mg/L—Longjiang River, 2012	[76,125,133]
**Lead** **Pb**	Increased incidence of histopathologies, Changes in number and morphology of germline cells, Up-regulation of expression of vitellogenin	4.6 ppb—mean concentration in surface waters, USA, 1996–2016	[124,134]

## Data Availability

Not applicable.

## References

[1] Jensen E.V., Jordan V.C. (2003). The estrogen receptor: A model for molecular medicine. Clin. Cancer Res..

[2] Kuiper G.G., Enmark E., Pelto-Huikko M., Nilsson S., Gustafsson J.A. (1996). Cloning of a novel receptor expressed in rat prostate and ovary. Proc. Natl. Acad. Sci. USA.

[3] Olde B., Leeb-Lundberg L.F. (2009). GPR30/GPER1: Searching for a role in estrogen physiology. Trends Endocrinol. Metab..

[4] Ignatov T., Modl S., Thulig M., Weißenborn C., Treeck O., Ortmann O., Ignatov A. (2013). GPER-1 acts as a tumor suppressor in ovarian cancer. J. Ovarian Res..

[5] Toran-Allerand C.D., Guan X., Maclusky N.J., Horvath T.L., Diano S., Singh M., Tinnikov A.A. (2002). ER-X: A novel, plasma membrane-associated, putative Estrogen Receptor that is regulated during development and after ischemic brain injury. J. Neurosci..

[6] Micevych P.E., Kelly J.M. (2021). Membrane estrogen receptor regulation of hypothalamic function. Neuroendocrinology.

[7] Prossnitz E.R., Barton M. (2011). The G-Protein-Coupled Estrogen Receptor GPER in health and disease. Nat. Rev. Endocrinol..

[8] Qiu J., Bosch M.A., Tobias S.C., Grandy D.K., Scanlan T.S., Rønnekleiv O.K., Kelly M.J. (2003). Rapid signaling of estrogen in hypothalamic neurons involves a novel G-Protein-Coupled estrogen receptor that activates protein kinase C. J. Neurosci..

[9] O’Lone R., Frith M.C., Karlsson E.K., Hansen U. (2004). Genomic targets of nuclear estrogen receptors. Mol. Endocrinol..

[10] Jacob J., Sebastian K.S., Devassy S., Priyadarsini L., Farook M.F., Shameem A., Thampan R.V. (2006). Membrane Estrogen Receptors: Genomic actions and post transcriptional regulation. Mol. Cell. Endocrinol..

[11] Lecomte S., Demay F., Ferrière F., Pakdel F. (2017). Phytochemicals targeting estrogen receptors: Beneficial rather than adverse effects?. Int. J. Mol. Sci..

[12] Acconcia F., Kumar R. (2006). Signaling regulation of genomic and nongenomic functions of estrogen receptors. Cancer Lett..

[13] Paris M., Pettersson K., Schubert M., Bertrand S., Pongratz I., Escriva H., Laudet V. (2008). An amphioxus orthologue of the estrogen receptor that does not bind estradiol: Insights into estrogen receptor evolution. BMC Evol. Biol..

[14] Hawkins M.B., Godwin J., Crews D., Thomas P. (2005). The distributions of the duplicate oestrogen receptors ER-βa and ER-βb in the forebrain of the Atlantic croaker (Micropogonias undulatus): Evidence for subfunctionalization after gene duplication. Proc. R. Soc. B: Biol. Sci..

[15] Pinto P.I., Andrade A.R., Estêvão M.D., Alvarado M.V., Felip A., Power D.M. (2018). Duplicated membrane estrogen receptors in the European sea bass (*Dicentrarchus labrax*): Phylogeny, expression and regulation throughout the reproductive cycle. J. Steroid Biochem. Mol. Biol..

[16] Booth A.N., Bickoff E.M., Kohler G.O. (1960). Estrogen-like activity in vegetable oils and mill by-products. Science.

[17] Ostrovsky D., Kitts W.D. (1962). Estrogen-Like substances in legumes and grasses: The influence of fractionation and route of administration on the estrogenic activity of plant materials. Can. J. Bioch. Physi..

[18] Bennetts H.W., Uuderwood E.J., Shier F.L. (1946). A specific breeding problem of sheep on subterranean clover pastures in western australia. Aust. Vet. J..

[19] Paterni I., Granchi C., Minutolo F. (2017). Risks and benefits related to alimentary exposure to xenoestrogens. Crit. Rev. Food Sci. Nutr..

[20] Alonso-Magdalena P., Ropero A.B., Soriano S., García-Arévalo M., Ripoll C., Fuentes E., Nadal Á. (2012). Bisphenol-A acts as a potent estrogen via non-classical estrogen triggered pathways. Molecul. Cell. Endocrin..

[21] Cao L.Y., Ren X.M., Guo L.H. (2019). Estrogen-related receptor Γ is a novel target for lower-chlorinated polychlorinated biphenyls and their hydroxylated and sulfated metabolites. Environ. Pollut..

[22] Lee D., Ko Y., Pang C., Ko Y.J., Choi Y.K., Kim K.H., Kang K.S. (2022). Estrogenic activity of mycoestrogen (3β, 5α, 22E)-Ergost-22-En-3-Ol via estrogen receptor α-dependent signaling pathways in MCF-7 cells. Molecules.

[23] Kinkade C.W., Rivera-Núñez Z., Gorcyzca L., Aleksunes L.M., Barrett E.S. (2021). Impact of fusarium-derived mycoestrogens on female reproduction: A systematic review. Toxins.

[24] Černá T., Ezechiáš M., Semerád J., Grasserová A., Cajthaml T. (2022). Evaluation of estrogenic and antiestrogenic activity in sludge and explanation of individual compound contributions. J. Hazard. Mater..

[25] Shirdel I., Kalbassi M.R., Esmaeilbeigi M., Tinoush B. (2020). Disruptive effects of nonylphenol on reproductive hormones, antioxidant enzymes, and histology of liver, kidney and gonads in caspian trout smolts. Compar. Biochem. Physio. Part C Toxicol. Pharmacol..

[26] Dobbs R.W., Malhotra N.R., Greenwald D.T., Wang A.Y., Prins G.S., Abern M.R. (2019). Estrogens and prostate cancer. Prostate Cancer Prostatic Dis..

[27] Gore A.C. (2007). Endocrine-Disrupting Chemicals: From Basic Research to Clinical Practice.

[28] Ronchetti S.A., Miler E.A., Duvilanski B.H., Cabilla J.P. (2013). Cadmium mimics estrogen-driven cell proliferation and prolactin secretion from anterior pituitary cells. PLoS ONE.

[29] Georgescu B., Georgescu C., Dărăban S., Bouaru A., Paşcalău S. (2011). Heavy metals acting as endocrine disrupters. Sci. Pap. Anim. Sci. Biotechnol..

[30] Chmielewski J., Łuszczki J., Czarny-Działak M., Dutkiewicz E., Król H., Gworek B., Nowak-Starz G. (2021). Environmental exposition to xenoestrogens and related health effects. J. Elem..

[31] Silva I.P., Brito D.C.C., Silva T.E.S., Silva R.F., Guedes M.I.F., Silva J.Y.G., Figueiredo J.R. (2021). In vitro exposure of sheep ovarian tissue to the xenoestrogens zearalenone and enterolactone: Effects on preantral follicles. Theriogenology.

[32] Ratajczak-Wrona W., Rusak M., Nowak K., Dabrowska M., Radziwon P., Jablonska E. (2020). Effect of bisphenol a on human neutrophils immunophenotype. Sci. Rep..

[33] Wang Y.Q., Li Y.W., Chen Q.L., Liu Z.H. (2019). Long-term exposure of xenoestrogens with environmental relevant concentrations disrupted spermatogenesis of zebrafish through altering sex hormone balance, stimulating germ cell proliferation, meiosis and enhancing apoptosis. Environ. Pollut..

[34] Torrens-Mas M., Roca P. (2020). Phytoestrogens for cancer prevention and treatment. Biology.

[35] Hsieh C.J., Hsu Y.L., Huang Y.F., Tsai E.M. (2018). Molecular mechanisms of anticancer effects of phytoestrogens in breast cancer. Curr. Protein Pept. Sci..

[36] Wang X., Ha D., Yoshitake R., Chan Y.S., Sadava D., Chen S. (2021). Exploring the biological activity and mechanism of xenoestrogens and phytoestrogens in cancers: Emerging methods and concepts. Int. J. Mol. Sci..

[37] Petrie B., Lopardo L., Proctor K., Youdan J., Barden R., Kasprzyk-Hordern B. (2019). Assessment of bisphenol-A in the urban water cycle. Sci. Total Environ..

[38] Samia K., Dhouha A., Anis C., Ammar M., Rim A., Abdelkrim C. (2018). Assessment of organic pollutants (PAH and PCB) in surface water: Sediments and shallow groundwater of grombalia watershed in northeast of tunisia. Arab. J. Geosci..

[39] Lu S., Lin C., Lei K., Xin M., Wang B., Ouyang W., He M. (2021). Endocrine-disrupting chemicals in a typical urbanized bay of yellow sea, china: Distribution, risk assessment, and identification of priority pollutants. Environ. Pollut..

[40] Luo Z., Tu Y., Li H., Qiu B., Liu Y., Yang Z. (2019). Endocrine-disrupting compounds in the Xiangjiang River of China: Spatio-temporal distribution, source apportionment, and risk assessment. Ecotoxicol. Environ. Saf..

[41] Radwan E.K., Ibrahim M.B.M., Adel A., Farouk M. (2020). The occurrence and risk assessment of phenolic endocrine-disrupting chemicals in egypt’s drinking and source water. Environ. Sci. Pollut. Res..

[42] Zhou X., Yang Z., Luo Z., Li H., Chen G. (2019). Endocrine disrupting chemicals in wild freshwater fishes: Species, tissues, sizes and human health risks. Environ. Pollut..

[43] Ribeiro C., Tiritan M.E., Rocha E., Rocha M.J. (2009). Seasonal and spatial distribution of several endocrine-disrupting compounds in the Douro River Estuary, Portugal. Arch. Environ. Contam. Toxicol..

[44] Yang S., Yu W., Yang L., Du B., Chen S., Sun W., Tang J. (2020). Occurrence and fate of steroid estrogens in a Chinese typical concentrated dairy farm and slurry irrigated soil. J. Agric. Food Chem..

[45] Wojnarowski K., Podobiński P., Cholewińska P., Smoliński J., Dorobisz K. (2021). Impact of estrogens present in environment on health and welfare of animals. Animals.

[46] Renaud L., Agarwal N., Richards D.J., Falcinelli S., Hazard E.S., Carnevali O., Hardiman G. (2019). Transcriptomic analysis of short-term 17α-ethynylestradiol exposure in two californian sentinel fish species sardine (*Sardinops Sagax*) and mackerel (*Scomber Japonicus*). Environ. Pollut..

[47] Meng Q., Yeung K., Kwok M.L., Chung C.T., Hu X.L., Chan K.M. (2020). Toxic effects and transcriptome analyses of zebrafish (*Danio Rerio*) larvae exposed to benzophenones. Environ. Pollut..

[48] Anderson J.C., Beyger L., Guchardi J., Holdway D.A. (2020). The effects of 17α-Ethinylestradiol on the heart rate of embryonic Japanese Medaka (Oryzias Latipes). Toxicol. Chem..

[49] Elvin T., Malini N., George K. (1793). Deleterious effect of short term exposure to xenoestrogen-bisphenol a on certain haematological and physiological profile of Freshwater Murrel. Channa Striata. Bloch.

[50] Kirk C.J., Bottomley L., Minican N., Carpenter H., Shaw S., Kohli N., Harris R.M. (2003). Environmental endocrine disrupters dysregulate estrogen metabolism and Ca^2+^ homeostasis in fish and mammals via receptor-independent mechanisms. Comp. Biochem. Physiol. Part A Mol. Integr. Physiol..

[51] Nelson E.R., Habibi H.R. (2013). Estrogen receptor function and regulation in fish and other vertebrates. Gen. Comp. Endocrinol..

[52] Sun S.-X., Wu J.-L., Lv H.-B., Zhang H.-Y., Zhang J., Limbu S.M., Du Z.-Y. (2020). Environmental estrogen exposure converts lipid metabolism in male fish to a female pattern mediated by AMPK And mtor signaling pathways. J. Hazard. Mater..

[53] Warner K.E., Jenkins J.J. (2007). Effects of 17α-Ethinylestradiol and bisphenol A on vertebral development in the fathead Minnow (*Pimephales Promelas*). Toxicol. Chem. Int. J..

[54] Zhang X., Zhong H., Han Z., Tang Z., Xiao J., Guo Z., Zhou Y. (2020). Effects of waterborne exposure to 17β-estradiol on hepatic lipid metabolism genes in Tilapia (*Oreochromis Niloticus*). Aquac. Rep..

[55] Zhang Y., Zhu Z., Liu Q., Zhang M., Yang H., Wei W. (2021). Bisphenol A disrupts Apolipoprotein e expression through estrogen-related receptor gamma and DNA methlylation in the liver of male rare Minnow (*Gobiocypris Rarus*). Ecotoxicol. Environ. Saf..

[56] Nilsson B.O., Olde B., Leeb-Lundberg L.F. (2011). G protein-coupled oestrogen receptor 1 (GPER1)/GPR30: A new player in cardiovascular and metabolic oestrogenic signalling. Br. J. Pharmacol..

[57] Thomas P., Alyea R., Pang Y., Peyton C., Dong J., Berg A.H. (2010). Conserved estrogen binding and signaling functions of the G Protein-coupled estrogen receptor 1 (GPER) in mammals and fish. Steroids.

[58] Teng J., Zhao Y., Chen H.J., Xue L.Y., Ji X.S. (2021). Global expression response of genes in sex-undifferentiated Nile tilapia gonads after exposure to trace letrozole. Ecotoxicol. Environ. Saf..

[59] Orozco-Hernández L., Gutiérrez-Gómez A.A., SanJuan-Reyes N., Islas-Flores H., García-Medina S., Galar-Martínez M., Gómez-Oliván L.M. (2018). 17β-Estradiol induces cyto-genotoxicity on blood cells of common carp (*Cyprinus carpio*). Chemosphere.

[60] Sator P.G., Schmidt J.B., Rabe T., Zouboulis C.C. (2004). Skin aging and sex hormones in women–clinical perspectives for intervention by hormone replacement therapy. Exp. Dermatol..

[61] Gill A., Patranabis S. (2021). Phytohormones as potential anticancer agents. Int. J. Res. Appl. Sci. Biotechnol..

[62] Sehmisch S., Hammer F., Christoffel J., Seidlova-Wuttke D., Tezval M., Wuttke W., Stuermer E.K. (2008). Comparison of the phytohormones genistein, resveratrol and 8-Prenylnaringenin as agents for preventing osteoporosis. Planta Med..

[63] Pham T.H., Page Y.L., Percevault F., Ferriere F., Flouriot G., Pakdel F. (2021). Apigenin, a partial antagonist of the estrogen receptor (ER), inhibits ER-Positive breast cancer cell proliferation through Akt/FOXM1 signaling. Int. J. Mol. Sci..

[64] Ruiz T.F.R., Colleta S.J., De Campos Zuccari D.A.P., Vilamaior P.S.L., Leonel E.C.R., Taboga S.R. (2021). Hormone receptor expression in aging mammary tissue and carcinoma from a rodent model after xenoestrogen disruption. Life Sci..

[65] Calaf G.M., Ponce Cusi R., Aguayo F., Muñoz J.P., Bleak T.C. (2020). Endocrine disruptors from the environment affecting breast cancer. Oncol. Lett..

[66] Henderson B.E., Feigelson H.S. (2000). Hormonal carcinogenesis. Carcinogenesis.

[67] Bohra A., Bhateja S. (2015). Carcinogenesis and sex hormones: A review. Endocrinol. Metab. Syndrome..

[68] Bhardwaj P., Au C.C., Benito-Martin A., Ladumor H., Oshchepkova S., Moges R., Brown K.A. (2019). Estrogens and breast cancer: Mechanisms involved in obesity-related development, growth and progression. J. Steroid Biochem. Mol. Biol..

[69] Jackson L., Klerks P. (2020). Effects of the synthetic estrogen 17α-Ethinylestradiol on heterandria formosa populations: Does matrotrophy circumvent population collapse?. Aquat. Toxicol..

[70] Karki N.P., Colombo R.E., Gaines K.F., Maia A. (2021). Exposure to 17β estradiol causes erosion of sexual dimorphism in bluegill (*Lepomis Macrochirus*). Environ. Sci. Pollut. Res..

[71] Lam S.H., Lee S.G., Lin C.Y., Thomsen J.S., Fu P.Y., Murthy K.R., Mathavan S. (2011). Molecular conservation of estrogen-response associated with cell cycle regulation, hormonal carcinogenesis and cancer in zebrafish and human. Cancer Cell Lines. BMC Med. Genom..

[72] Chaturantabut S., Shwartz A., Evason K.J., Cox A.G., Labella K., Schepers A.G., Goessling W. (2019). Estrogen Activation of G-Protein–coupled estrogen receptor 1 regulates phosphoinositide 3-Kinase and Mtor signaling to promote liver growth in zebrafish and proliferation of human hepatocytes. Gastroenterology.

[73] Roy D., Mitra A., Homechaudhuri S. (2021). Differential expression of genes responsible for reproduction of male and female *Danio rerio* following interaction between estrogen receptor and cadmium. Proc. Zool. Soc. Springer India.

[74] Brama M., Gnessi L., Basciani S., Cerulli N., Politi L., Spera G., Migliaccio S. (2007). Cadmium induces mitogenic signaling in breast cancer cell by an Erα-dependent mechanism. Mol. Cell. Endocrinol..

[75] Stoica A., Katzenellenbogen B.S., Martin M.B. (2000). Activation of estrogen receptor—A by the heavy metal Cadmium. Mol. Endocrinol..

[76] Kim Y.J., Lee N., Woo S., Ryu J.C., Yum S. (2016). Transcriptomic change as evidence for Cadmium-induced endocrine disruption in marine fish model of Medaka, *Oryzias Javanicus*. Mol. Cell. Toxicol..

[77] Christiansen T., Korsgaard B., Jespersen A. (1998). Effects of nonylphenol and 17 beta-oestradiol on vitellogenin synthesis, testicular structure and cytology in male eelpout Zoarces viviparus. J. Exp. Biol..

[78] Azevedo D.D.A., Lacorte S., Viana P., Barceló D. (2001). Occurrence of nonylphenol and bisphenol-A in surface waters from Portugal. J. Braz. Chem. Soc..

[79] Ganesan S., Keating A.F. (2016). Bisphenol A-induced ovotoxicity involves DNA damage induction to which the ovary mounts a protective response indicated by increased expression of proteins involved in DNA repair and xenobiotic biotransformation. Toxicol. Sci..

[80] Khan N.G., Correia J., Adiga D., Rai P.S., Dsouza H.S., Chakrabarty S., Kabekkodu S.P. (2021). A comprehensive review on the carcinogenic potential of bisphenol A: Clues and evidence. Environ. Scicience Pollut. Res..

[81] Dumitrascu M.C., Mares C., Petca R.C., Sandru F., Popescu R.I., Mehedintu C., Petca A. (2020). Carcinogenic effects of bisphenol a in breast and ovarian cancers. Oncol. Lett..

[82] Cervantes-Camacho I., Guerrero-Estévez S.M., López M.F., Alarcón-Hernández E., López-López E. (2020). Effects of Bisphenol a on Foxl2 gene expression and DNA damage in adult viviparous fish *Goodea Atripinnis*. J. Toxicol. Environ. Health Part A.

[83] Major K.M., Decourten B.M., Li J., Britton M., Settles M.L., Mehinto A.C., Brander S.M. (2020). Early life exposure to environmentally relevant levels of endocrine disruptors drive multigenerational and transgenerational epigenetic changes in a fish model. Front. Mar. Sci..

[84] Callard G.V., Tchoudakova A.V., Kishida M., Wood E. (2001). Differential tissue distribution, developmental programming, estrogen regulation and promoter characteristics of Cyp19 genes in teleost fish. J. Steroid Biochem. Mol. Biol..

[85] Filice M., Leo S., Mazza R., Amelio D., Garofalo F., Imbrogno S., Gattuso A. (2021). The heart of the adult goldfish (*Carassius auratus*) as a target of bisphenol A: A multifaceted analysis. Environ. Pollut..

[86] Moreman J., Takesono A., Trznadel M., Winter M.J., Perry A., Wood M.E., Tyler C.R. (2018). Estrogenic mechanisms and cardiac responses following early life exposure to bisphenol A (BPA) and its metabolite 4-Methyl-2, 4-Bis (P-Hydroxyphenyl) Pent-1-Ene (MBP) in zebrafish. Environ. Sci. Technol..

[87] Bakos K., Kovács R., Staszny Á., Sipos D.K., Urbányi B., Müller F., Kovács B. (2013). Developmental toxicity and estrogenic potency of zearalenone in zebrafish (*Danio Rerio*). Aquat. Toxicol..

[88] Amenyogbe E., Chen G., Wang Z., Lu X., Lin M., Lin A.Y. (2020). A review on sex steroid hormone estrogen receptors in mammals and fish. Int. J. Endocrinol..

[89] Budczies J., Brockmöller S.F., Müller B.M., Barupal D.K., Richter-Ehrenstein C., Kleine-Tebbe A., Fiehn O. (2013). Comparative metabolomics of estrogen receptor positive and estrogen receptor negative breast cancer: Alterations in glutamine and beta-alanine metabolism. J. Proteom..

[90] Loomis A.K., Thomas P. (2000). Effects of estrogens and xenoestrogens on androgen production by Atlantic croaker testes in vitro: Evidence for a nongenomic action mediated by an estrogen membrane receptor. Biol. Reprod..

[91] Mohapatra S., Chakraborty T., Shimizu S., Ohta K., Nagahama Y., Ohta K. (2020). Estrogen and estrogen receptors chauffeur the sex-biased autophagic action in liver. Cell Death Differ..

[92] Tanida I., Ueno T., Kominami E. (2008). LC3 and autophagy. Methods Molecural Biol..

[93] Feswick A., Munkittrick K.R., Martyniuk C.J. (2017). Estrogen-responsive gene networks in the teleost liver: What are the key molecular indicators?. Environ. Toxicol. Pharmacol..

[94] Knopp R.H., Paramsothy P., Retzlaff B.M., Fish B., Walden C., Dowdy A., Cheung M.C. (2006). Sex differences in lipoprotein metabolism and dietary response: Basis in hormonal differences and implications for cardiovascular disease. Curr. Cardiol. Rep..

[95] Cleveland B.M., Weber G.M. (2016). Effects of steroid treatment on growth, nutrient partitioning, and expression of genes related to growth and nutrient metabolism in adult triploid rainbow trout (*Oncorhynchus Mykiss*). Domest. Anim. Endocrinol..

[96] Sovadinová I., Liedtke A., Schirmer K. (2014). Effects of clofibric acid alone and in combination with 17β-Estradiol on mRNA abundance in primary hepatocytes isolated from rainbow trout. Toxicol. Vitr..

[97] Celino-Brady F.T., Petro-Sakuma C.K., Breves J.P., Lerner D.T., Seale A.P. (2019). Early-Life exposure to 17β-Estradiol and 4-Nonylphenol impacts the growth hormone/insulin-like growth-factor system and estrogen receptors in Mozambique Tilapia, *Oreochromis Mossambicus*. Aquat. Toxicol..

[98] Müller A.K., Markert N., Leser K., Kämpfer D., Schiwy S., Riegraf C., Hollert H. (2021). Bioavailability and impacts of estrogenic compounds from suspended sediment on rainbow trout (*Oncorhynchus mykiss*). Aquat. Toxicol..

[99] Breves J.P., Duffy T.A., Einarsdottir I.E., Björnsson B.T., McCormick S.D. (2018). In vivo effects of 17α-ethinylestradiol, 17β-estradiol and 4-nonylphenol on insulin-like growth-factor binding proteins (igfbps) in Atlantic salmon. Aquat. Toxicol..

[100] Lazaro-Velasco A., Isidro-Cristobal H.M., Alcántar-Vázquez J.P., Antonio-Estrada C., Calzada-Ruiz D., Torre R.M.D.L. (2019). Effect of the combination of a cold-water temperature and exogenous estrogens on feminization, growth, gonadosomatic index and fat muscle content of Nile tilapia *Oreochromis niloticus* (Linnaeus, 1758). Lat. Am. J. Aquat. Res..

[101] Kovats S. (2015). Estrogen receptors regulate innate immune cells and signaling pathways. Cell. Immunol..

[102] Cunningham M., Gilkeson G. (2011). Estrogen receptors in immunity and autoimmunity. Clin. Rev. Allergy Immunol..

[103] Iwanowicz L.R., Ottinger C.A. (2009). Estrogens, estrogen receptors and their role as immunoregulators in fish. Fish Def..

[104] Chaves-Pozo E., García-Ayala A., Cabas I. (2018). Effects of sex steroids on fish leukocytes. Biology.

[105] Seemann F., Knigge T., Duflot A., Marie S., Olivier S., Minier C., Monsinjon T. (2016). Sensitive periods for 17β-estradiol exposure during immune system development in Sea Bass Head kidney. J. Appl. Toxicol..

[106] Moreira C., Paiola M., Duflot A., Varó I., Sitjà-Bobadilla A., Knigge T., Monsinjon T. (2021). the influence of 17β-oestradiol on lymphopoiesis and immune system ontogenesis in juvenile Sea Bass, *Dicentrarchus Labrax*. Dev. Comp. Immunol..

[107] Woźny M., Obremski K., Hliwa P., Gomułka P., Różyński R., Wojtacha P., Brzuzan P. (2019). Feed contamination with zearalenone promotes growth but affects the immune system of rainbow trout. Fish Shellfish Immunol..

[108] Abdel-Tawwab M., Khalil R.H., Diab A.M., Khallaf M.A., Abdel-Razek N., Abdel-Latif H.M., Khalifa E. (2021). Dietary garlic and chitosan enhanced the antioxidant capacity, immunity, and modulated the transcription of HSP70 and cytokine genes in zearalenone-intoxicated european Seabass. Fish Shellfish Immunol..

[109] Park K., Kwak I.S. (2013). Expression of Stress Response HSP70 Gene in Asian Paddle Crabs, *Charybdis Japonica*, Exposure to endocrine disrupting chemicals, bisphenol A (BPA) and 4-Nonylphenol (NP). Ocean Sci. J..

[110] Li X., Wang S., Qi J., Echtenkamp S.F., Chatterjee R., Wang M., Gupta D. (2007). zebrafish peptidoglycan recognition proteins are bactericidal amidases essential for defense against bacterial infections. Immunity.

[111] Stamenkovic I., Yu Q., Merlin A. (2010). “Magic” linker between the extracellular cues and intracellular signaling pathways that regulate cell motility, proliferation, and survival. Curr. Protein Pept. Sci..

[112] Abdel-Tawwab M., Khalifa E., Diab A.M., Khallaf M.A., Abdel-Razek N., Khalil R.H. (2020). Dietary garlic and chitosan alleviated zearalenone toxic effects on performance, immunity, and challenge of european sea bass, *Dicentrarchus Labrax*, to *Vibrio Alginolyticus* infection. Aquac. Int..

[113] Rehberger K., Von Siebenthal E.W., Bailey C., Bregy P., Fasel M., Herzog E.L., Segner H. (2020). Long-Term exposure to low 17α-ethinylestradiol (EE2) concentrations disrupts both the reproductive and the immune system of juvenile rainbow trout, *Oncorhynchus Mykiss*. Environ. Int..

[114] Kernen L., Phan A., Bo J., Herzog E.L., Huynh J., Segner H., Baumann L. (2022). Estrogens as immunotoxicants: 17α-ethinylestradiol exposure retards thymus development in zebrafish (*Danio Rerio*). Aquat. Toxicol..

[115] Liu S., Chen F., Zhang Y., Cai L., Qiu W., Yang M.G. (2021). protein-coupled estrogen receptor 1 mediates estrogen effect in red common carp (*Cyprinus carpio*). Comp. Biochem. Physiol. Part C Toxicol. Pharmacol..

[116] Maciuszek M., Pijanowski L., Pekala-Safinska A., Kemenade B.M., Chadzinska M. (2020). 17β-Estradiol affects the innate immune response in common carp. Fish Physiol. Biochem..

[117] Maciuszek M., Pijanowski L., Pekala-Safinska A., Palichleb P., Błachut M., Verburg-van Kemenade B.L., Chadzińska M. (2020). 17α-ethinylestradiol and 4-tert-octylphenol concurrently disrupt the immune response of common carp. Fish Shellfish Immunol..

[118] Wang Y.L., Zhou X.Q., Jiang W.D., Wu P., Liu Y., Jiang J., Feng L. (2019). Effects of dietary zearalenone on oxidative stress, cell apoptosis, and tight junction in the intestine of juvenile grass carp (*Ctenopharyngodon idella*). Toxins.

[119] Pacheco Passos Neto O., Bezerra dos Santos A., Feitosa Silva J.R., Mota S. (2021). Alterations in the development and gonadal structure of Nile Tilapia (*Oreochromis niloticus*) exposed to natural and synthetic estrogens. Water Air Soil Pollut..

[120] Fan X., Hou T., Jia J., Tang K., Wei X., Wang Z. (2020). Discrepant dose responses of bisphenol A on oxidative stress and DNA methylation in grass carp ovary cells. Chemosphere.

[121] Forner-Piquer I., Fakriadis I., Mylonas C.C., Piscitelli F., Di Marzo V., Maradonna F., Carnevali O. (2019). Effects of dietary bisphenol A on the reproductive function of gilthead sea bream (*Sparus aurata*) testes. Int. J. Mol. Sci..

[122] Xie Q.P., Li B.B., Wei F.L., Yu M., Zhan W., Liu F., Lou B. (2021). Growth and gonadal development retardations after long-term exposure to estradiol in little yellow croaker, *Larimichthys polyactis*. Ecotoxicol. Environ. Saf..

[123] Voisin A.S., Kültz D., Silvestre F. (2019). Early-life exposure to the endocrine disruptor 17-α-ethinylestradiol induces delayed effects in adult brain, liver and ovotestis proteomes of a self-fertilizing fish. J. Proteom..

[124] Łuszczek-Trojnar E., Drąg-Kozak E., Szczerbik P., Socha M., Popek W. (2014). Effect of long-term dietary lead exposure on some maturation and reproductive parameters of a female Prussian carp (*Carassius gibelio B*.). Environ. Sci. Pollut. Res..

[125] Paschoalini A.L., Savassi L.A., Arantes F.P., Rizzo E., Bazzoli N. (2019). Heavy metals accumulation and endocrine disruption in *Prochilodus argenteus* from a polluted neotropical river. Ecotoxicol. Environ. Saf..

[126] Pojana G., Gomiero A., Jonkers N., Marcomini A. (2007). Natural and synthetic endocrine disrupting compounds (EDCs) in water, sediment and biota of a coastal lagoon. Environ. Int..

[127] Tang Z., Liu Z.H., Wang H., Dang Z., Liu Y. (2021). A review of 17α-ethynylestradiol (EE2) in surface water across 32 countries: Sources, concentrations, and potential estrogenic effects. J. Environ. Manag..

[128] Rotimi O.A., Olawole T.D., De Campos O.C., Adelani I.B., Rotimi S.O. (2021). Bisphenol A in Africa: A review of environmental and biological levels. Sci. Total Environ..

[129] Gromadzka K., Waśkiewicz A., Goliński P., Świetlik J. (2009). Occurrence of estrogenic mycotoxin–zearalenone in aqueous environmental samples with various NOM content. Water Res..

[130] Jin X., Jiang G., Huang G., Liu J., Zhou Q. (2004). Determination of 4-tert-octylphenol, 4-nonylphenol and bisphenol A in surface waters from the Haihe River in Tianjin by gas chromatography–mass spectrometry with selected ion monitoring. Chemosphere.

[131] Domagalski J.L., Weston D.P., Zhang M., Hladik M. (2010). Pyrethroid insecticide concentrations and toxicity in streambed sediments and loads in surface waters of the San Joaquin Valley, California, USA. Environ. Toxicol. Chem. Int. J..

[132] King O.C., van de Merwe J.P., McDonald J.A., Leusch F.D. (2016). Concentrations of levonorgestrel and ethinylestradiol in wastewater effluents: Is the progestin also cause for concern?. Environ. Toxicol. Chem..

[133] Zhao X.M., Yao L.A., Ma Q.L., Zhou G.J., Wang L., Fang Q.L., Xu Z.C. (2018). Distribution and ecological risk assessment of cadmium in water and sediment in Longjiang River, China: Implication on water quality management after pollution accident. Chemosphere.

[134] Frank J.J., Poulakos A.G., Tornero-Velez R., Xue J. (2019). Systematic review and meta-analyses of lead (Pb) concentrations in environmental media (soil, dust, water, food, and air) reported in the United States from 1996 to 2016. Sci. Total Environ..

